# The Multilevel Limb‐loss and Preservation Rehabilitation Continuum model (MLPRC): An evidence‐based implementation model integrating multiple perspectives to improve outcomes for people facing limb loss

**DOI:** 10.1002/pmrj.13300

**Published:** 2025-01-30

**Authors:** Prateek Grover, Elvin Geng, Rachel G. Tabak

**Affiliations:** ^1^ Penn State Hershey Medical Center and Penn State College of Medicine Hershey Pennsylvania USA; ^2^ Washington University in St. Louis St. Louis Missouri USA

## Abstract

**Background:**

The patient journey from threatened to actual limb loss, subsequent rehabilitation, and limb preservation through the health system is complicated and prone to delays, attrition, and inequities. A shared multi(socioecological)‐level conceptualization of this journey among the multiple stakeholders is the first step for systematically improving limb care through identification of bottlenecks and gaps, contributory factors, and responsive interventions and implementation strategies.

**Objective:**

To meet this knowledge gap by presenting a novel implementation model, the Multilevel Limb‐loss and Preservation Rehabilitation Continuum (MLPRC), that can be used to develop multilevel applications.

**Methods:**

MLPRC was developed in three overlapping steps: (1) design of the patient journey through the health system (horizontal element), (2) addition of multilevel perspectives along the journey (vertical element), and (3) implementation facilitation by incorporating implementation model constructs (concentric cells element).

**Results:**

MLPRC is an implementation model for limb loss and preservation with three concentric cells: *inner context cell* (horizontal time‐ordered patient journey at patient level, based on phases of amputation rehabilitation and patient journey concepts, and corresponding care delivery at provider/organization‐level, based on the continuum of care and lower limb loss rehabilitation continuum concepts, on the journey timeline), less influenceable *outer context cell* (community, system and policy level perspectives), and *outermost implementation cell*, based on Proctor Model of Implementation Research, that utilizes *inner and outer context* cells (concept derived from Exploration, Preparation, Implementation, Sustainment framework) information to define responsive metrics, interventions and implementation strategies.

**Discussion:**

Examples of MLPRC use as a blueprint for multilevel applications include *patient level* (education and navigation instruments), *provider level* (integrated practice clinic, referral streams), *organization level* (limb care continuum programs), and *community*, *system*, and *policy level* (interdisciplinary organizational partnerships, data repositories).

**Conclusion:**

MLPRC is among the first implementation model to present both a unified picture of the field of limb loss and preservation and a blueprint for multilevel applications.

## INTRODUCTION

Worldwide, 180 million people are living with limb loss, with 5.5 million years lived with disability.^1^ In addition, global prevalence of major causes of threatened limb loss such as peripheral vascular disease and diabetic foot ulcer approximates 236 million^2^ and 33 million,^3^ respectively. Traditionally, care for the former is provided by limb loss rehabilitation programs and for the latter by limb preservation programs. Given that many people with limb loss, especially from a dysvascular etiology, are at risk for subsequent limb loss, it makes sense for limb loss rehabilitation programs to partner with limb preservation programs, as well as with mental health and medical risk management (eg, primary care to manage diabetes) programs.^4^ This comprehensive care approach can be used to envision the multidisciplinary field of limb loss and preservation. Using the concept of the patient journey (journey) through the health system, this field can be further defined as care for the journey preceding an amputation (limb preservation) and for the journey from amputation to initial functional independence and subsequent maintenance of function and prevention of limb loss (limb loss rehabilitation).

This field experiences major health care disparities related to factors such as race, insurance, and geographic location, with better characterization for limb preservation^5^ compared with limb loss rehabilitation. To better understand and appropriately address these health inequities through implementation strategies such as competency‐based care provider training, care delivery pathways, health services research, and policy translation,^6^ it is vital to first understand the field in a comprehensive and coherent manner. However, conceptualizing this field in a unified fashion for responsive implementation is challenging.

One reason a unified conceptualization is challenging is the heterogeneity of the patient journey from limb loss to independence in the community, influenced by factors at multiple socioecological levels. Patient expectations, experience, and care needs can vary with multilevel factors such as the cause of limb loss,^7^, ^8^ access to structured limb care programs, connection with community resources, and systems of care.^9^ A second reason is the multidisciplinary yet siloed nature of the field, which results in overlapping but differing priorities, terminology, and metrics to characterize the patient journey and outcomes.^10^, ^11^ This does not allow the stakeholders to align and work together as effectively as might be possible. A third reason is the complex multistep nature of the patient journey within the health system, which is better characterized in countries with a centralized health care system such as Canada.^12^ Overall, there is a lack of clear definition of these steps for operational purposes to guide program development and refinement.

Hence, the field needs a model that unifies the complex, multistep patient journey through the health system with multilevel perspectives for analysis of influential factors, and with implementation science principles to create a shared conceptualization of progress, identify bottlenecks and gaps, define responsive strategies addressing those gaps, and implement, refine, and sustain interventions. Such a comprehensive Multilevel Limb‐loss and Preservation Rehabilitation Continuum Model (MLPRC) would be useful for patient enablement, professional stakeholder collaboration, health care organization program development, and structured evidence‐generation for policy through theory‐grounded research. The aim of this article is to present such a model developed through a synthesis of established literature‐based concepts from health care administration, rehabilitation, and implementation science and to highlight some potential applications with basic guidelines.

## METHODS

**FIGURE 1:**
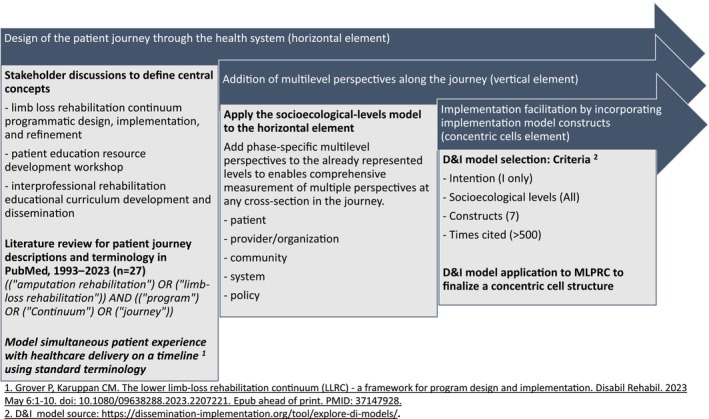
Multilevel limb‐loss and preservation rehabilitation continuum model design steps: (1) horizontal element design of the patient journey through the health system, (2) vertical element addition to incorporate diverse perspectives along the journey, and (3) concentric cell structure integration to facilitate implementation. ^1^ Grover P, Karuppan CM. The lower limb‐loss rehabilitation continuum (LLRC)—a framework for program design and implementation. *Disabil Rehabil*. 2023; 46(8): 1652–1661. doi: 10.1080/09638288.2023.2207221 . ^2^ D&I model source: https://dissemination‐implementation.org/tool/explore‐di‐models/ . Abbreviations: D&I, dissemination and implementation; MLPRC, Multilevel Limb‐loss and Preservation Rehabilitation Continuum.

Design of this implementation model for the field of limb loss and preservation, the MLPRC, involved three overlapping steps: (1) design of the patient journey through the health system (horizontal element), (2) addition of multilevel perspectives along the journey (vertical element), and (3) implementation facilitation by incorporating implementation model constructs (concentric cells element) (Figure 1).

The author, P.G., with clinical care, leadership, educational resource design and development, and program implementation experience in the field of limb loss and preservation rehabilitation completed steps 1 and 2, and 2 senior authors, R.T. and E.G., as experienced dissemination and implementation (D&I) scientists, guided step 3. The author, P.G., then applied the model to create a blueprint for potential applications, some of which are presented in the discussion section.

### Design of the patient journey through the health system (horizontal element)

Concepts central to the limb loss journey through the health care continuum were identified through literature reviews and multiple formal and informal stakeholder discussions over 6 years related to limb loss systems development and implementation, but not explicitly focused on model development. Stakeholder discussion settings included (1) limb loss rehabilitation continuum programmatic design, implementation, and refinement in three health systems (Mercy Clinic, Springfield, MO, 2017–2019; Washington University, St Louis, MO, 2019–2023; Penn State Health, Hershey, PA, 2023–present), (2) patient education resource development workshop (Amputee Coalition First Step manual, 7th edition, Washington, DC, 2019), and (3) interprofessional rehabilitation educational curriculum development and dissemination (American Congress of Rehabilitation Medicine Limb Care Networking Group Instructional Courses 2020, 2021). The central concepts identified were *amputation*/*limb loss rehabilitation programs*, *patient journeys*, and *continuums of care*.

Using these concepts, a literature review was last conducted in PubMed in December 2023 with the search strategy: ((“*amputation rehabilitation*”) *OR* (“*limb‐loss rehabilitation*”)) *AND* ((*program*) *OR* (*Continuum*) *OR* (*journey*)), limited to articles from past 30 years, with no limit on article type to retrieve literature on limb loss program description. In total, 27 articles were retrieved and abstracts reviewed; 17 articles with predominant clinical outcomes, surgical, or biomechanical focus, and one article unrelated to amputation were excluded. The remaining seven articles with a predominant program focus were reviewed for limb loss rehabilitation program and patient journey description. Four articles described the Amputation Systems of Care and Transdisciplinary Amputation Care Team Approach (TACT),^9^, ^13^, ^14^, ^15^ with most defining four phases of rehabilitation, namely, perioperative, preprosthetic, prosthetic training, and lifelong care. One article each described the nine phases of amputation rehabilitation,^10^ the lower limb loss rehabilitation continuum implementation framework (LLRC)^16^ with five steps of care delivery and a LLRC timeline, and a socioecological level‐based limb care structural model.^4^ Phase, step, and timeline concepts from these models were used to define the horizontal time‐ordered journey‐continuum for visualizing, measuring, and describing the simultaneous patient experience with health care delivery on a shared timeline.

### Addition of multilevel perspectives along the journey (vertical element)

The socioecological‐levels model^4^ was used to provide additional vertical phase‐specific multilevel perspectives at community, system, and policy levels to the initial structure of the horizontal time‐ordered journey‐continuum that already represented patient and provider/organization levels. This step enables comprehensive measurement of multiple perspectives at any cross‐section in the journey.

### Implementation facilitation by incorporating implementation model constructs (concentric cells element)

To integrate preimplementation and implementation approaches into the horizontal structure, D&I models were reviewed and selected from the Dissemination and Implementation Models in Health interactive webtool^17^ (Table 1).

**TABLE 1:** Implementation model and framework selection methodology for concentric cell structure integration to facilitate implementation of the Multilevel Limb‐Loss and Preservation Rehabilitation Continuum model.

S. no	Implementation models	Socioecological levels	Constructs	Cited	Field of origin
1	Consolidated framework for implementation research	I, O, C, S, P	C, H, S	2980	Health services
2	Active implementation framework	I, O, C	B, St, S	1870	Education
3	Proctor's implementation outcomes	I, O, C, S, P	C, St	1130	Mental health
4	Exploration, preparation, implementation, sustainment (EPIS) model	I, O, C, S, P	B, D, St, S	898	Public sector services
5	Weiner organizational readiness	I, O, C	B	796	Organizational psychology
6	Conceptual model of implementation research	I, O, C, S	St	541	Mental health

*Note*: Socioecological levels: Individual, I; Organization, O; Community, C; System, S; Policy, P. Constructs: Barriers and facilitators, B; Development of an Intervention, D; Stakeholders, St; Strategies, S; Cost, C; Health Equity, H; Outcomes, O.

Selection criteria included intention (implementation), socioecological levels (individual, organization, community, system, policy), constructs (barriers and facilitators, cost, development of an implementation, health equity, outcomes, stakeholders, strategies), and times cited (more than 500 times). Six models met most of the criteria: Consolidated Framework for Implementation Research (CFIR); Active Implementation Framework; Proctor's Implementation Outcomes; Exploration, Preparation, Implementation, Sustainment (EPIS) model; Weiner organizational readiness; and Conceptual Model of Implementation Research (Table 1). Three of the six models (CFIR, Proctor's Implementation Outcomes, and EPIS) included all five socioecological levels.^27^ Of these three, EPIS included four constructs (barriers and facilitators, development of an intervention, stakeholders, strategies), followed by CFIR, that included two constructs (cost, health equity). EPIS and Proctor's Implementation Outcomes model concepts were then used to finalize a concentric cell structure.

## RESULTS

**FIGURE 2:**
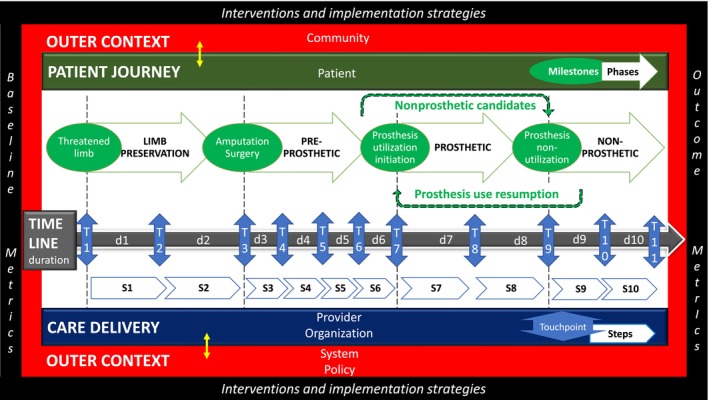
The Multilevel Limb‐Loss and Preservation Rehabilitation Continuum model (MLPRC). Structure: (1) inner context cell (white)—horizontal time‐ordered journey progression; (2) outer context cell (red)—includes community, system, and policy socioecological levels, and (3) outermost implementation cell (black)—provides a mechanism for utilization of inner and outer context cells information. Standard terminology: Milestones (m)—discrete events of change in the patient journey related to their limb that change their functional status. Phase (p)—time periods between milestones that reflect changes in their overall experience based on the major functional status change. Touchpoints (t)—identifiable points of interaction between the patient and the health system and are labeled by a date on the timeline. Step (s)—periods of care delivery between touchpoints. Durations (d)—time spent by the patient within a step, calculated for each step by subtracting origin touchpoint date from end touchpoint date. Abbreviation: PM&R, physical medicine and rehabilitation.

The model, as a combination of “limb‐loss and preservation rehabilitation continuum” and “multilevel factors,” was labeled the MLPRC model, with design elements, terminology, and model description presented next (Figure 2).

### Design elements

The first of three design elements is horizontal time‐ordered journey‐continuum progression of limb‐loss and preservation rehabilitation, quantified on a timeline derived from the quality improvement tools such as run charts.^18^ The second design feature is vertical phases‐specific multilevel perspectives at patient, provider, organization, community, system, and policy levels, derived from socioecological levels.^19^ The third is a concentric structure, with *inner and outer context cells* based on EPIS,^20^ and *outermost implementation cell* based on the Proctor Model of Implementation Research.

The *inner context cell* follows the horizontal progression of the *patient journey* (patient socioecological‐level) based on phases of amputation rehabilitation^10^, ^21^ and patient journey concepts,^22^ and corresponding *care delivery* (provider and organization socioecological levels) based on the continuum of care^23^ and LLRC^24^ concepts, on the *journey timeline*. This level is more directly impactable by an organization. The *outer context cell* extends to include community, system, and policy socioecological levels, and is often more difficult to influence directly. The *outermost implementation cell* provides a mechanism for utilization of inner and outer context cells information for preimplementation and implementation applications. This includes socioecological level‐specific baseline metrics characterization at the beginning of the patient journey that should lead to identification of feasible outcome metrics, interventions and implementation strategies, and ultimately postimplementation achievement of desired outcomes.

Double headed arrows on the timeline and between inner and outer context cells reflect vertical integration/interaction between levels. They also serve as a cross‐sectional timepoint view to study the socioecological‐level specific status at that time‐point of characteristics, interventions and implementation strategies, and outcomes.

### Terminology

The MLPRC patient journey includes two standard terms, milestone and phases, defined using the limb, rehabilitation, and prosthetic device as central themes of the patient experience.^25^ Milestones (m) are discrete events of change in the patient journey related to their limb that change their functional status. These include date of threatened limb, amputation surgery, prosthesis receipt, and prosthesis use cessation. Phases (p) are time periods between milestones that reflect changes in their overall experience based on the major functional status change. The four sequential phases between the four aforementioned milestones are *limb preservation*, *preprosthetic phase*, *prosthetic phase*, and *nonprosthetic phase*. It is important to note that not all persons with limb loss will experience all milestones and phases. As an example, not all patients will be prosthetic device candidates and may stay in the preprosthetic phase. Similarly, people in the prosthetic phase may never cease device use, staying in this phase.

*MLPRC care delivery* includes two standard terms, touchpoints and steps.^24^ Touchpoints (t) are identifiable points of interaction between the patient and the health system^26^ and represent the organization perspective. Examples include dates of surgery, admission to and discharge from inpatient preprosthetic rehabilitation, functioning evaluation and prescription, prosthetic device receipt, initiation and completion of prosthetic rehabilitation. Steps (s) refer to periods of care delivery between touchpoints. Each step can occur at distinct or overlapping institutions, with their own structure, personnel, and processes. Examples include postsurgical stabilization, preprosthetic rehabilitation—inpatient or outpatient or combination, prosthetic fitting, prosthetic rehabilitation—inpatient or outpatient or combination, and prosthetic change. Gaps between steps in the model highlight the potential gaps in transition of care between steps that should be paid attention to and minimized. Ideally, from a program development application perspective, all milestones should also be touchpoints for accurate patient journey mapping, but this may not occur related to lack of recall, examples being date of wound development or ceasing prosthesis use.

The MLPRC timeline is made up of touchpoints (dates) and intervening durations. Durations (d) identify the time spent by the patient within a step. For each step, durations in days are calculated by subtracting origin touchpoint date from end touchpoint date. When many touchpoints occur in a very short span of time, such as multiple surgeries or multiple clinic visits for prosthesis fitting, it is recommended to choose the most reliable and meaningful ones to minimize data collection overload.

### Model description

#### Inner context cell

Description of the patient journey, care delivery, and timeline will vary depending on a combination of patient, care providers, and organization factors. Phase and milestone definitions are intended to be standard, retained terms in the model that ensure inclusion of the patient experience. Timepoints and step definitions are intended to be adaptable by care providers and organizations specific to their resources and goals (Table 2).

**TABLE 2:** Multilevel Limb‐Loss and Preservation Rehabilitation Continuum model inner context cell illustration of patient journey milestone and phase, and health care delivery touchpoint and step examples for clinical use.

	Phases	Limb preservation phase	Preprosthetic phase	Prosthetic phase	Nonprosthetic phase
Patient experience	Phase focus examples	Limb loss prevention Shared decision making for surgical options	Function with assistive devices (wheelchair, walker, etc.) Prosthetic device appropriateness Rehabilitation setting evaluation	Training and use of prosthetic device Device modifications over time	Function with assistive devices (wheelchair, walker, etc.) Applies to both nonprosthetic candidates and prosthetic device use cessation
	Milestone examples	Threatened limb to amputation surgery	Amputation surgery to prosthesis receipt	Prosthesis receipt to prosthesis use cessation	No prosthesis utilization
Care delivery	Touchpoint examples	Threatened limb Healed limb	Surgery Admission to preprosthetic rehabilitation	Device receipt Admission to prosthetic rehabilitation	Nonprosthetic use determination abandonment of device
	Step examples	Community‐level function while wound is healing	Postsurgical stabilization	Device receipt to rehabilitation	Nonprosthetic level functioning
Multilevel factors/Determinants	Patient‐level examples	Etiology of threatened limb	Limb healing	Prosthesis related goals	Medical conditions limiting prosthesis use
	Organization‐level examples	Access to surgical services	Access to preprosthetic rehabilitation	Access to prosthetic and community rehabilitation	Access to medical care for modifying risk factors and mental health

The limb preservation phase lies between *milestones* of threatened limb and surgery. The *patient journey* focuses on saving the limb and difficult decisions regarding various surgical and salvage options. *Touchpoint* examples are care episodes for threatened limb, healed limb, surgical decision‐making, nonamputation surgery, and amputation surgery. *Steps* can be defined to describe intervening community‐level functioning while wound is healing and presurgical planning. *Durations* depend on factors such as cause of threatened limb and care accessed.

The preprosthetic phase lies between *milestones* of amputation surgery and prosthesis device utilization initiation. The *patient journey* focuses on functioning with assistive devices while the residual limb heals and receipt of a prosthetic device, if appropriate. *Touchpoint* examples are surgery, admission and discharge to preprosthetic rehabilitation, wheelchair and assistive device evaluation and receipt, functioning evaluation and prescription, and prosthetic device receipt. *Step* examples are postsurgical stabilization, preprosthetic rehabilitation, limb healing and maturation, and prosthetic fitting. *Durations* depend on a combination of patient factors such as healing and system factors such as close coordination.

The prosthetic phase lies between *milestones* of prosthesis utilization initiation and prosthesis non‐utilization. The *patient journey* focuses on initial training with the prosthetic device, device modifications over time to address functioning and device needs, and continuing to function with the prosthetic device until no longer possible. *Touchpoints* examples are device receipt, admission and discharge to prosthetic rehabilitation, and subsequent cyclical functioning evaluation and prescription with device receipt. *Step examples* are device receipt to rehabilitation, prosthetic rehabilitation, and then cyclical prosthetic‐level functioning and prosthetic fitting. *Durations* depend on a combination of factors such as residual limb and device changes over time, patient participation goals and performance, and additional access to community rehabilitation services such as driving and vocational and recreational rehabilitation.

The nonprosthetic phase lies beyond *milestone* of prosthesis nonutilization. The *patient journey* focuses on continued functioning without a prosthetic device and includes nonprosthetic candidates as well as prosthetic device users who are no longer using a prosthetic device. This phase can be permanent, such as when device use is limited by perceived utility or risk, or temporary, such as an acute health condition limiting use until the person resumes device use to enter back into the prosthetic phase. *Touchpoint* examples would be nonprosthetic use determination, abandonment of device medical, functioning, wheelchair and assistive device evaluation and receipt, and prosthetic device evaluation(s). S*tep examples* would include nonprosthetic level functioning and prosthetic fitting. *Durations* depend on the cause of prosthetic device abandonment and subsequent medical course and patient goals.

#### Outer context cell

Community factors such as access to peer‐mentors (community level), limb loss care systems such as the Veterans Health Administration Amputation Systems of care (system level), and policy factors such as health care funding mechanisms (policy level) influence the patient journey,^27^ care delivery, and timeline. Purposeful understanding of these levels and their impact at different phases and steps can be conducted using the implementation cell to enable optimal utilization of available resources and creation of missing resources.

#### Implementation cell

*Baseline metrics* should be defined at each socioecological level to understand the baseline status including patient demographics and equity challenges, provider and system resources, and external context, to help identify barriers/determinants and feasible outcome metrics. *Outcome metrics* in the patient, service, and implementation domains, and *interventions* responsive to barriers should be defined for each socioecological level. *Implementation strategies* can then be defined from a compilation of implementation strategy tools such as Expert Recommendations for Implementing Change (ERIC).^6^

The implementation project can then be planned, implemented, refined, and sustained using implementation science tools such as EPIS^20^ and/or health care administration tools such as Strategic Thinking Maps.^28^

## DISCUSSION

The discussion starts with contrasting MLPRC with other limb loss and preservation models and highlighting the former's comparative benefit as an implementation science model for developing and implementing multilevel applications. Theories, models, and frameworks utilized for MLPRC design are described next for a broad audience of clinicians, researchers, administrators, and implementation scientists to elaborate on source concepts from diverse fields that were vital to synthesize the MLPRC. The discussion concludes with illustrative applications of the MLPRC.

### Comparing MLPRC with other models for limb loss and preservation

The MLPRC is a novel implementation model that conceptualizes the field of limb loss and preservation and offers a succinct mechanism to design and implement a multilevel packet of interventions and implementation strategies responsive to contextual barriers and facilitators.

Other comparative models include the TACT algorithm and the LLRC framework.^16^ TACT is widely utilized within the Veterans' Health Amputation Systems of Care, but most organizations do not have the knowledge and resources to successfully replicate this multilevel national program. The LLRC, in contrast, was developed in a nonacademic semiurban setting and has broader applicability. The LLRC can be considered as a subset of the MLPRC, and they differ in several significant ways. The MLPRC is an overarching model of the myriad limb loss and preservation journeys, whereas the LLRC focuses only on the lower limb loss journey. The MLPRC fits the definition of an implementation science “model” with process model elements, as it describes the process of translation into practice. The LLRC is an implementation “framework” with evaluation framework elements as it helps to evaluate implementation. Both contain elements of determinant frameworks as they both seek to understand factors that affect implementation outcomes.^29^ The MLPRC structure includes three cells, whereas the LLRC includes only part of the systems perspective of the horizontal continuum. By virtue of its structure, MLPRC applications extend beyond program implementation to several others such as patient education and navigation tools, interprofessional education and coordination, organizational partnerships and registry development. The LLRC, as a framework, is primarily intended for program implementation and evaluation for the lower limb journey only.

### Description of theories, models, and frameworks utilized for MLPRC design

#### Health care administration concepts

The patient journey concept is utilized to understand the patient experience as they travel through the health system, often for an episode of care,^22^ in order to refine their health system, frequently in synergy with other project management tools.^30^ This established concept has been used as the central theme by national patient advocacy organizations such as the Amputee Coalition for designing their patient resource manual for limb loss, the *First Step: AGuide for Adapting to Limb Loss*.^31^ The continuum of care concept^23^ is useful for development of continuum‐of‐care programs, and is widely used not only in the field of rehabilitation^32^ but also other fields such as oncology^33^ and maternal and child health.^34^ The conceptual definition can be used to derive principles for systems development, including (1) systems of care that are integrated, (2) health services that are comprehensive, (3) care provision that extends over time and care settings, and (4) structure that facilitates patient navigation and monitoring. These principles are especially important for limb loss and preservation rehabilitation care delivery, because the patient journey following limb loss is complicated and requires close coordination between the patient, care teams, and organizations for optimal functioning. Quality improvement run charts are established tools to track changes in metrics over time^18^ and offer the opportunity to quantify the progress of a person with limb loss over the course of their journey.

#### Rehabilitation science concepts

The patient experiences after amputation surgery have been described as uncertainty with functional ability, rehabilitation, and prosthetic devices.^25^ Patient journeys with limb loss described in literature, although differing in the span of the patient journey, typically focus on surgery along with one or more of these vital patient experience elements. The most granular description includes nine phases, with the first three focusing on surgery (preoperative, amputation surgery/reconstruction, and acute postsurgical), the next three on rehabilitation and prosthetic device (preprosthetic, prosthetic prescription, and prosthetic training), and the last three on function in the community (community integration, vocational rehabilitation, and follow‐up).^10^ Five quantitative phases have been described in a study on falls with the first three focusing on the care episode from surgery to completion of rehabilitation (postoperative‐**‐*/ period, preprosthetic phase, prosthetic rehabilitation), and the fourth and fifth focusing on function with a prosthetic device (new normal phase) and after ceasing prosthetic device use (postprosthetic phase), respectively.^21^ The Veterans Association Transdisciplinary Amputation Care Team approach reduces the number of phases to four, with the first focused on surgery (perioperative), the next two on rehabilitation and prosthetic device (preprosthetic, prosthetic training), and the fourth on function (lifelong care).^1^ The five‐ and nine‐phase approaches have been used to create a five‐step limb loss rehabilitation program implementation framework for lower limb loss, the LLRC, that spans the patient journey and care episode from surgery to completion of prosthetic rehabilitation.^24^

#### Implementation science models

Socioecological levels are an accepted mechanism for understanding and addressing public health issues such as the Centers for Disease Control and Prevention's (CDC's) violence prevention,^35^ as well as rehabilitation conditions such recovery after stroke and associated factors.^36^ These levels have been adopted for program implementation as well.^37^ These levels can vary in number from four to six, depending on the application, and include a concentric hierarchical structure. As an example, the CDC model levels include the individual at the core, followed by relationship, community, and societal levels (https://www.cdc.gov/violenceprevention/about/social-ecologicalmodel.html). This multilevel perspective can be useful to create a structured method to identify level‐specific baseline and outcome metrics as well as interventions and implementation strategies. The Proctor Model of Implementation Research defines interventions, implementation strategies, and outcomes as key components of implementation research methods. Interventions are evidence‐based practices that can be applied to facilitate change. Implementation strategies are methods to facilitate intervention application, modification, and long‐term use. Outcomes include implementation, service, and client outcomes categories.^38^ The EPIS framework^20^ is an implementation framework of four phases, namely, Exploration, Preparation, Implementation, and Sustainment that uses four constructs, outer context, inner context, bridging factors, and innovation factors. The inner context focuses on organization and person level characteristics, which could be considered representative of individual, care team, and organization socioecological levels. The outer context includes interorganizational networks, funding and service environment, which can be considered representative of community, system, and policy levels. As the socioecological‐levels framework is useful for limb loss and preservation program development^4^ and has been used as one of the three design features of MLPRC, mapping this framework to EPIS allows for a practical method for model users to apply EPIS tools to MLPRC.

### Illustrative applications of MLPRC: multilevel implementation blueprint

The MLPRC can be considered as an evidence‐based intervention, and its illustrative applications of the model can be considered as multilevel strategies, discussed subsequently (Table 3).

**TABLE 3:** Summary of some Multilevel Limb‐Loss and Preservation Rehabilitation Continuum model (MLPRC) applications—multilevel implementation blueprint.

Socioecological levels	Evidence‐based interventions	Guidelines for implementation
Inner context		
Patient level	Patient progress card (Figure 3).	Create a simple diagram of the patient journey, with ideal timeline. Add space for the patient/provider to enter actual dates. Add details of providers, institutions, and community resources for each step.
Provider level	Interdisciplinary education and coordination workshop.	Create a simple diagram of the patient journey, with ideal timeline. Use it to discuss and develop coordination mechanisms such as integrated practice clinics, joint inpatient rounding, and referral streams between providers.
Organization level	Limb loss rehabilitation continuum (LLRC) program.	Create a simple diagram of common patient journeys in your community, with ideal timeline (eg, lower limb dysvascular). Create a program metrics set comprised of inner context baseline and outcomes metrics that are meaningful and can be easily collected at timepoints or within steps. Collect and analyze the data to identify barriers, intervention, and implementation strategies. Create a project charter. Use implementation science and/or health care administration methods to implement, refine, and sustain.
Outer context		
Community, system, and policy level	Community organization partnerships. Interdisciplinary curriculum. Data repositories.	Define outer context socioecological‐level characteristics to identify potential resources, barriers, interventions and implementation strategies. Conduct stakeholder meetings and establish resources using implementation science/health care administration methods.

#### Patient level

**FIGURE 3:**
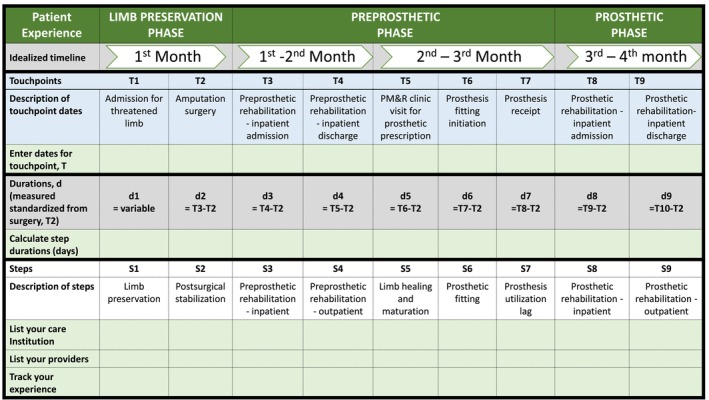
Illustration of Multilevel Limb‐Loss and Preservation Rehabilitation Continuum model (MLPRC) structure and standard terminology utilization for patient‐level applications—MLPRC Patient Progress Card. The light green boxes are for patient utilization, and the others represent underlying model structure and terminology.

For patient education, a simple diagram can be created for discussion using phases and milestone of patient journey, and touchpoints and steps of care delivery. To enable ease of navigation of the system, details of providers, institutions, and community resources for each step can be added. To additionally monitor progress and enable patient inclusion in decision‐making, a patient progress card can be created with an ideal timeline and space for the patient/provider to enter actual dates, convert to step durations to compare with ideal timeline, and keep track of step and phase institutions, providers, and experiences (Figure 3).

#### Provider level

For provider education, a diagram focusing on inner context, including phases of patient journey, milestones, touchpoints and steps of care delivery, and timeline can be created. To promote coordination, the discussion to outer context and implementation can be conducted in a workshop format, with provider and resource assessment using standard questionnaires and tools such as Capability, Opportunity, and Motivation‐Behavior (COM‐B) Model for Behavior Change, and consensus on implementation of context‐appropriate coordination mechanisms such as integrated practice clinics, joint inpatient rounding, and referral streams between providers.

#### Organization level

Common types of patient journey (such as traumatic upper limb loss and dysvascular lower limb loss) touchpoints, steps of care delivery, and an ideal timeline can be defined to create journey‐specific frameworks. Inner context socioecological‐level baseline metrics at entry into the journey as well as socioecological‐level outcomes that are meaningful and can be easily collected at timepoints or within steps can then be defined and combined to create the program metrics set. Data can be collected and analyzed to identify barriers, interventions in addition to the LLRC (system‐level intervention), and implementation strategies. Project charter can then be created followed by implementation refinement and sustainment through utilization of implementation science tools such as EPIS and/or health care administration tools such as health care strategic maps.

#### Community, system, and policy level

Outer context socioecological‐level characteristics can be defined to identify potential resources, barriers, interventions, and implementation strategies. Examples of strategies include collaboration for community resources such as peer‐mentors and limb loss care systems, exploring continuum replication and sustainment opportunities, especially if part of or partnering with national or regional level specialty care providers such as for postacute rehabilitation or prosthetics. To influence policy level, cost‐effectiveness analyses using data generated by continuum of care programs can be conducted with results disseminated to policymakers.

## LIMITATIONS

Although there was informal stakeholder input from various disciplines including physical therapy, occupational therapy, orthotics and prosthetics, plastic surgery, trauma surgery, orthopedics, podiatry, implementation scientists, and people with lived experiences, clinical aspects of model development were conducted by one author, a clinician–scientist with expertise in limb loss and preservation rehabilitation. Institutional review board‐approved focus group format stakeholder engagement was not part of methods. Additionally, the model was not informed by a systematic review of the literature. Only one database was searched, which may limit the articles that informed the model. As the MLPRC is the first effort to succinctly conceptualize the field of limb loss and preservation, it is hence intended to be a living model that will evolve based upon feedback from stakeholders as well from insight in implementing the applications. Future work will include a formal review of the model by stakeholders and clinical experts in the field for model refinement.

## CONCLUSION

The MPPRC is a living model for the field of limb loss and preservation rehabilitation that unifies complex multilevel perspectives on the patient journey with limb loss through a complicated health system to create a shared conceptualization of progress, identify bottlenecks and gaps, and identify strategies addressing those gaps. This heuristic can provide the field with a shared understanding of goals, coherent metrics of success and as an organizing scaffolding for strategies that act at various socioecological levels and steps in the continuum. The model is intended for care providers, organizations, researchers and policymakers to adapt and utilize for improving care and outcomes for persons with limb loss and threatened limb loss.

## DISCLOSURES

The authors declare no financial conflicts of interest (COI). Potential nonfinancial COI include Prateek Grover—Limb Care Networking Group, American Congress of Rehabilitation Medicine—Chair; Quality, Policy, Practice and Research Committee, American Academy of Physician Medicine and Rehabilitation—Member at Large; Orthotic and Prosthetic Outcomes Research Program, Congressionally Directed Medical Research Program—Programmatic Reviewer; Scientific and Medical Advisory Committee, Amputee Coalition—Member.
